# Integrated health service delivery networks and tuberculosis avoidable hospitalizations: is there a relation between them in Brazil?

**DOI:** 10.1186/s12913-016-1320-y

**Published:** 2016-03-01

**Authors:** Marcela Paschoal Popolin, Michelle Mosna Touso, Mellina Yamamura, Ludmila Barbosa Bandeira Rodrigues, Maria Concebida da Cunha Garcia, Luiz Henrique Arroyo, Antônio Carlos Vieira Ramos, Thais Zamboni Berra, Marcelino Santos Neto, Juliane de Almeida Crispim, Francisco Chiaravalotti Neto, Ione Carvalho Pinto, Pedro Fredemir Palha, Severina Alice da Costa Uchoa, Luís Velez Lapão, Inês Fronteira, Ricardo Alexandre Arcêncio

**Affiliations:** Maternal-Infant and Public Health Nursing Department, College of Nursing of Ribeirão Preto, University of São Paulo, Av dos Bandeirantes 3900, 14040-902 Ribeirão Preto, São Paulo Brazil; Institute for Health Sciences, Federal University of Mato Grosso, Av Alexandre Ferronato 1200, Reserve 35, 78550-000 Sinop, Mato Grosso Brazil; Centre of Social Sciences, Health and Technology of the Federal University of Maranhão (UFMA), Rua Turqueza, 65900-410 Imperatriz, Maranhão Brazil; Department of Epidemiology, Faculty of Public Health, University of São Paulo, Avenida Dr. Arnaldo, 715, 01246-904 São Paulo, São Paulo Brazil; Department of Group Health, Federal University of Rio Grande do Norte, Avenida Senador Salgado Filho, 3000, Rio Grande do Norte 59078-970 Natal, Brazil; WHO Collaborating Centre for Health Workforce Policy and Planning, International Public Health and Biostatistics, Global Health and Tropical Medicine, Instituto de Higiene e Medicina Tropical, Universidade Nova de Lisboa, Rua Junqueira 100, Lisbon, P-1349-008 Portugal; International Public Health and Biostatistics, Global Health and Tropical Medicine, Instituto de Higiene e Medicina Tropical, Universidade Nova de Lisboa, Rua Junqueira 100, Lisbon, P-1349-008 Portugal

**Keywords:** Primary Health Care, Health Care Network coordination, Tuberculosis, Hospitalization, Spatial analysis

## Abstract

**Background:**

The early identification of the Breathing Symptoms within the scope of Primary Health Care is recommended, and is also one of the strategies of national sanitary authorities for reaching the elimination of tuberculosis. The purpose of this study is to consider which attributes and which territories have shown the most significant progress in Primary Health Care, in terms of coordination of Health Care Networks, and also check if those areas of Primary Health Care that are most critical regarding coordination, there were more or less cases of avoidable hospitalizations for tuberculosis.

**Methods:**

This is an ecological study that uses primary and secondary data. For analysis, coropletic maps were developed through the ArcGIS software, version 10.2. There was also the calculation of gross annual and Bayesian rates for hospitalizations for tuberculosis, for each Primary Health Care territory.

**Results:**

There were satisfactory results for attributes such as Population (*n* = 37; 80.4 %), Primary Health Care (*n* = 43; 93.5 %), Support System (*n* = 45; 97.8 %); the exceptions were Logistics System (*n* = 32; 76.0 %) and Governance System, with fewer units in good condition (*n* = 31; 67.3 %). There is no evidence of any connection between networks’ coordination by Primary Health Care and tuberculosis avoidable admissions.

**Conclusion:**

The results show that progress has been made regarding the coordination of the Health Care Networks, and a positive trend has been shown, even though the levels are not excellent. It was found no relationship between the critical areas of Primary Health Care and tuberculosis avoidable hospitalizations, possibly because other variables necessary to comprehend the phenomena.

## Background

The year of 2015 starts with an audacious goal set by the World Health Organization (WHO), to eliminate tuberculosis (TB) completely by the year 2050 [1]. In 2013 alone, the world saw over 9 million cases of the disease identified, and within this universe only 6 million had the possibility to access health services. Brazil is on the list of 22 countries that account for 80 % of all TB cases in the world, being in 22^nd^ position when it comes to incidence with 46 cases per 100,000 inhabitants, and a prevalence of 57 cases per 100,000 inhabitants [1].

Also in this group, Brazil is one of the countries with the lowest percentage of patients completing their treatment (72 %), well below the targets defined by the WHO, of 85 % [1], which puts the country in a situation of risk, especially because of multidrug resistance to tuberculosis (MDR-TB), which is when the patient becomes resistant to Rifampicin and Isoniazid. Among the expectations for the period after 2015, one is that the country could make progress in terms of the reorganization of its systems for ensuring access to the populations affected by the disease, especially the vulnerable segments.

Early identification of the Breathing Symptoms within the scope of Primary Health Care (PHC) is recommended, and is also one of the strategies used by national sanitary authorities to reach the expected target, which is the elimination of TB [2]. Counting on PHC for the coordination of care for the TB patient could have a significant effect in terms of completion of the course of treatment, and also in the breakage of the transmission chain, thanks to the reinforcement of the actions for promotion of health, prevention, treatment and rehabilitation, among others.

However, recent studies have shown an increase in admittances to hospital with TB [3, 4]; a transversal cut study in the municipality of São Paulo, comparing the outcomes between patients hospitalized with those receiving outpatient treatment, showed a lower rate of cure and a higher rate of mortality among the patients who had been hospitalized [5]. Another study, this one of an ecological type, performed in the state of São Paulo [6] showed a prevalence of hospitalizations among males, and also the presence of a significant number of hospitalized children, and clearly shows that PHC has been quite feeble in promoting health, prevention of new cases, diagnosis and early treatment.

There is a movement around the world towards the strengthening of the health system through PHC, with acknowledgement of its strategic function, so important for the organization of an integrated system, or a system in Networks [7]. The Pan-American Health Organization (PAHO) [8, 9] has recommended that Latin American and Caribbean countries organize their health systems using the logic of the Health Care Networks (HCNs), coordinated by the PHC, understanding that the way in which they are currently organized has not contributed to a solution of the main health problems, while also producing sanitary indicators that are a cause for concern and hospitalizations that could be avoided, at very high costs which cannot be borne by countries of low to medium income.

Even though there is not one single organizational model for the HCNs coordinated by the PHC, evidence [9, 10] shows that a set of attributes is essential for the operation thereof, namely: *Population and territories* that are well defined, and in which there should be a significant knowledge of preferences and needs, which ends up being a determining factor in the supply of health services; *Primary Health Care*, which is a model for health care comprising a multiprofessional team which covers the whole population, being an entry point while integrating and coordinating health care; a *Support System*, which consists of systems for diagnostic and therapeutic support, information systems, and systems for tele-assistance; a *Logistic System* which is provided by the institution of electronic registration, access and regulation systems, and also the transport systems; and a *Governance System* which has been created for the whole Network, in order to establish and implement unique mission, vision and strategies for the health services that are part of this system.

We feel that a health event is avoidable when a system has all the resources or technologies available in the health area to solve the problems or even to make sure these do not occur; however, as a result of faults in the distribution system, allocation of resources, organization and supply of health actions, they end up occurring [11]. In this way, the PHC also has the social role of equalizing the offer of actions resulting from health demands of the population and under this logic, the systems raise their capacity to solve the problems and hence become more efficient and equitative [12].

In Latin America, reorganization centred on the coordination of care by PHC is still in its early stages. The history of segmentation and fragmentation of most of the Latin American systems, with an offer of selective PHC, have weakened such aspects as the establishment of integrated networks and the continuation of health care [13]. Even though the attributes that are of decisive importance for the conformation of an HCN and the coordination of health care are accepted and acknowledged, we have not observed any studies which have considered these elements for a more pragmatic appraisal of PHC and not even that they have analysed the indicators of morbidity from TB, from this perspective.

As the territorial areas should be the unit of intervention of PHC and the structure of an HCN should be organized considering the local and regional conditions of these areas, as also the respective population [9, 10, 12], it is important to invest in new approaches that manage to bring the unit of space, also as a study object.

Even though the Geographical Information System (GIS) has not been widely used for the assessment of the health systems, this is a very interesting resource and could truthfully represent the social reality of the territories, in their interface with health service systems [14]. A systematic review revealed that GIS is a potentially powerful assessment tool for the assessment of the quality of PHC and can help in describing and explaining disparities in access and health outcomes [15].

Considering that the configuration of a health system in an HCN is an essential condition for the truthfulness and ability to solve problems, of the health services, and thus for the control of TB in Brazil, and also the fact that there are decisive or strategic characteristics for effective coordination by PHC, we propose to investigate the issue of where, and in which attributes, the PHC has made most progress in terms of coordination of the HCN in a large Brazilian municipality, and also check to see whether there has been an increase in avoidable hospitalizations due to TB in the critical areas of PHC.

## Methods

### Study design

This is an ecological and exploratory study [16] that has considered the territorial areas gathered with the PHC as a unit of analysis.

### Scenery

The study took place in the city of Ribeirão Preto, State of São Paulo, Brazil, in an area of some 650 km^2^ and an estimated population of 658,059 inhabitants, with about 99.7 % of this population residing in urban areas [17].

It is a city of high demographic density, and is included in the group of cities that does not present favourable social indicators, instead of showing high degree of wealth [18]. Regarding the epidemiological indicators of TB, it is stressed that Ribeirão Preto is on the list of priority municipalities for the control of this disease, in 2013 reaching an incidence of 28.2 cases per 100,000 people and also a percentage of cure equal to 77.8 % [19].

Regarding the geo-politico-administrative organization of the HCN, the municipality is divided into five Health Districts (North, South, Central, East and West), with differences regarding the population living in each region, ranging from 92,568 people in the South District to 173,908 people in the East District. PHC is distributed among 46 territorial areas, of which 16 (34.7 %) have Family Health Teams, these teams comprising, at the very least, one general practitioner (GP) or Family doctor, a generalist nurse, a nursing assistant or technician, and community health agents [20]. In 2013, the populational coverage provided by the PHC teams was 51.0 % and the proportion of Hospitalizations for Conditions Sensitive to Primary Care (ICSAP) was 18.9 % [19].

### Sources of data

Primary and secondary data was used to respond to the goals of the study. The primary data was obtained between January and December 2014 through interviews with the health professionals, making use of a special instrument drawn up by Mendes [21] and validated by researchers from the College of Nursing at Ribeirão Preto, part of the University of São Paulo [22].

In this study, the Hospital Information System (known as SIH, the abbreviation in Portuguese), a system that records all cases of avoidable hospitalizations resulting from tuberculosis, is also used as a source of data.

### Population and sample

In the first phase of the study, the population was taken as the 1334 health professionals, of University and high school level, who work in the PHC of the municipality. A minimum non-proportional and stratified random sample was also established, as a result of heterogeneity in the number of workers, considering the different Health Districts [23], considering a level of confidence of 95 %, with a tolerable sampling error of 5 %, statistical power of the test equal to 80 %, and a proportion (p) of 50 % of professionals who coordinate health care at the HCNs; thus a minimum sample of seven people was defined for each of the 46 units of PHC in the municipality of Ribeirão Preto.

Regarding secondary data, there was a study of avoidable cases of admittance to hospital of patients with TB, referring to codes A15.0 to A17.9 on the International Classification of Diseases (ICD-10), representing the clinical manifestations of TB that are sensitive to Primary Health Care, according to the laws and normative rulings of Brazil [24] between 2006 and 2012.

### Instruments of measurement

In this study, a questionnaire was used to assess Networks, consisting of 78 structured questions, using a five-point Likert scale, with responses of total disagreement (1); disagreement (2); neutral opinion (3), agreement (4) and total agreement (5), and also comprising five different attributes according to Table 1.

**Table 1:** Attributes and their items to assess progress of the PHC in terms of coordination of the HCN

Attribute of the instrument for data collection	Essence of content of items**	Number of items of the instrument, by dimension	Nature of the Variable	Scale
Population	Lives in single sanitary territories, with social organization into families, and being recorded and filed into subpopulations based on social and sanitary risks.	14	Politomic	Likert – Five-point rating scale (from total disagreement to total agreement).
Primary Health Care	RAS Communications Centre, through which all the services offered by the network communicate with each other	19		
Support System	These are the institutional venues where services common to all health care points are offered, in the fields of diagnostic support and therapy, pharmaceutical assistance and also the health information systems.	15		
Logistic System	These are technological solutions, anchored to information Technologies, which ensure that there is an organization of regulation of access, throughout the points of health care and support systems, in the networks.	16		
Governance System	This is the organizational or pluriinstitutional arrangement that allows the management of all the components of the RAS, so as to generate a co-operative excess between the social players in a situation that could increase the interdependence between them and lead to good health and economic results for the adstricted population.	14		

Note: (**Adapted from Rodrigues et al. [25])

To characterize the health professional of the study, the variables considered were professional category, sex, age, time with the institution, and work schedule. As for TB available hospitalizations, the authors considered two variables: sex and age.

For this study, there was also consideration of the cartographic base of the PHC territorial areas, made available by the Company for the Economic Development of Ribeirão Preto (CODERP) and also the base of the digital road system, StreetBase Basic*®* acquired from the company Imagem®.

### Analysis of data

The primary research data was entered in duplicate and in an independent manner. In the beginning, descriptive statistics was applied using the software Statistica version 7.9, with a counting of the categorical variables (sex) and also the calculation of measurements of central tendency (mean and median) for continuous variables, which are age of the professional and of the case of hospitalization, time of professional with the institution; these variables were categorized based on the median.

To assess progress of the PHC in terms of coordination of the HCN, the authors considered the data obtained through application of the tool to the health professionals. During the process of analysis, the scores (Sc) were duly calculated, by attributed score or globality, using the following formula:

$$ Sc=\frac{{\displaystyle \sum it}}{Np}\times 200 $$

Where ∑*it* is the sum of responses to all Likert items for each attribute or all attributes (globality) and N is number of health professionals (Np) from the PHC facility who participated of the study. For each PHC facility, a score was calculated and health unit was classified in relation to the quartiles, adopting the criteria: Between 0 and 25 %, *unsatisfactory condition of the PHC* in that attribute or with the attribute considered globally in coordination of the HCNs; 25.01 to 50 %, *fairly good condition* of the PHC; 50.01 to 75 %, *good condition* of the PHC; and 75.01 to 100 %, *excellent condition* of the PHC [26].

Next, coropletic maps were constructed, with the classification of globality and also by attributes, making use of the ArcGIS software, version 10.2. In possession of the maps, the next step was the geocoding of the avoidable hospitalizations as a result of TB. At this stage, it was necessary to standardise the addresses according to the digital road system base in a ‘shapefile’ file, with a SIRGAS 2000 UTM Zone 23S projection, then proceeding with the process of association of table data to a map which is incorporated into a SIG environment with the use of the TerraView software, version 4.2.2.

As not all the addresses have been found on the cartographic database, we made use of the Batch Geocode and Findlatitudeandlongitude packages, to help with the process of geocodification [27]. Later, there was the calculation of the raw annual rate of admittances to hospital for each territory of PHC, considering the numerator as being the total number of hospitalizations which occurred in the 7 years (*Yi*) and the denominator being the population residing in the territory (*Pi*) in the year 2012. The rates thus obtained were multiplied by 100 000 and the results thus obtained were then multiplied by a factor of 1/7, to obtain the mean annual rate of hospitalization by PHC territory, based on the formula [28]:

$$ T{b}_i=\frac{Y_i}{P_i}\times \frac{1}{7}\times 100,000 $$

After obtaining the annual rate, these were subjected to the mitigation process caused by the use of the empirical Bayesian model, with the aim of reducing the so-called oscillations of small numbers and also the events of interest based on the sub-register [29]. As a result of the application of the method, we have obtained a weighted average between the raw rates of the PHC territories and the rate of the region of the closest neighbours, taken as a reference. For these analyses we used the Terraview software, version 4.2.2. Next, there was generation of the map with the distribution of the local empirical Bayesian rates, grouped into quintiles using the ArcGIS software, version 10.2.

Multiple linear regression was then performed, using the method of least squares and also the strategy known as Stepwise Forward Selection, using the same attributes as for the Networks (Table 1) as independent variables and the annual gross rate of avoidable hospitalizations for TB as dependent, backed up by the criterion of the choice of the best explicative model, based on the highest value of adjusted R2 [30].

### Ethical considerations

The study was approved by the Research Ethics Committee of the School of Nursing of Ribeirão Preto, of the University of São Paulo (EERP/USP), in compliance with Ruling No. 466/2012 of the National Health Council, and also of the Regulating Standards and Guidelines for research involving human subjects, under protocol number 20036113.9.000.5393.

## Results

Table 2 shows the characteristics of 355 health professionals who have been recruited for the study, observing that 220 (81.7 %) were female, 184 (51.8 %) were under 46 years of age, and there was a prevalence of workers with a high-school education, with 89 (25.1 %) being Nursing Assistants or Technicians and 83 % (23.4 %) being Community Health Agents (CHAs).

**Table 2:** Social and demographic characteristics of the health professionals working in Primary Health Care, Ribeirão Preto, São Paulo, Brazil (2014)

Variables	n (355)	%
Sex		
Male	65	18.3
Female	290	81.7
Age		
≤46 years	184	51.8
>46 years	171	48.2
Professional Category		
Nursing Assistant and Technician	89	25.1
CHA	83	23.4
Doctor	49	13.8
Nurse	45	12.7
Other Technicians	44	12.4
Dentist	29	8.1
Other University-level professional people	16	4.5
Years in the position		
≤5	99	27.9
5 – 10	90	25.3
10.1 – 15	58	16.3
15.1 – 20	39	11.1
>20	69	19.4

We also noticed that 99 (27.9 %) of the workers have been associated to PHC services for less than 5 years, while 69 (19.4 %) are associated for over 20 years.

We identified 169 cases of avoidable hospitalizations through TB, which works out at an average rate of 26.2 cases per 100,000 people. We could also observe that most hospitalizations were among males (*n* = 134, 79.3 %) with a mean and median age of 48 years, a minimum age of 6 and a maximum of 98 years, with a standard deviation (SD) of 16.2 years.

**Fig. 1:**
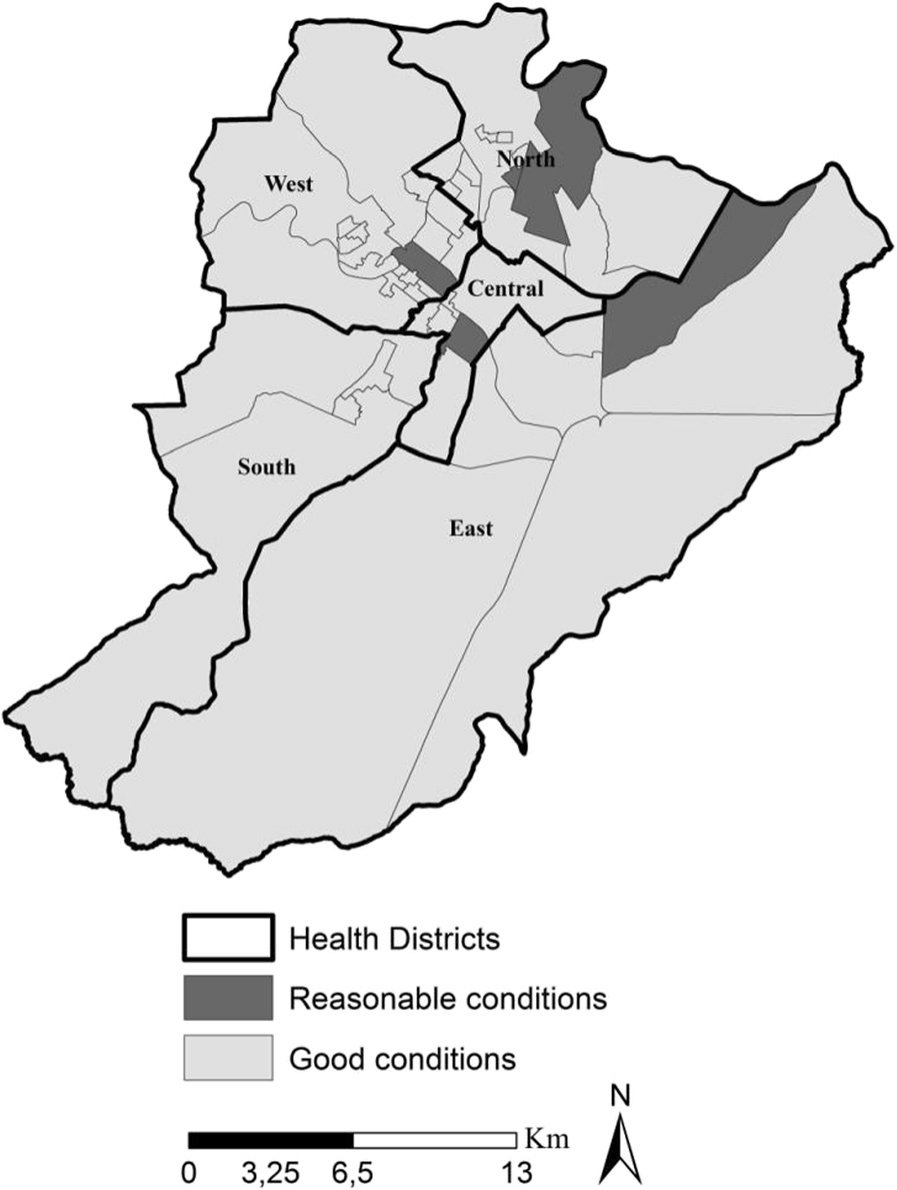
Classification of Primary Health Care, according to the capacity of coordination, Ribeirão Preto, São Paulo, Brazil (2014)

Figure 1 brings the classification of the PHC units, observing the fact that five had reasonable conditions for the coordination of health care and the others showed good conditions.

**Fig. 2:**
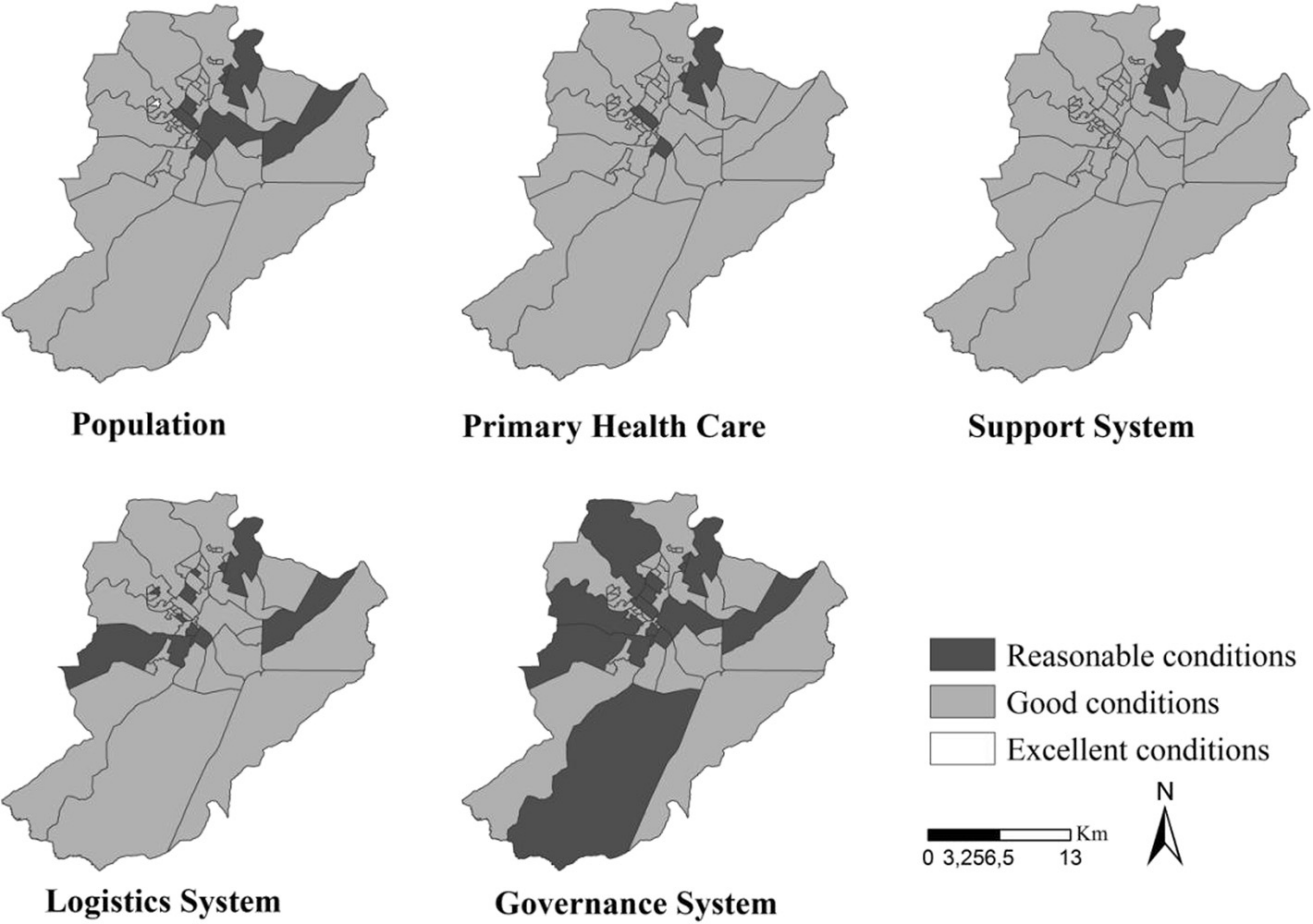
Assessment of Primary Health Care following the attributes for coordination of Networks and territories of Primary Health Care, Ribeirão Preto, São Paulo, Brazil (2006–2014)

Figure 2 shows the classification of the territories of the PHC units, according to the HCN attributes. We see that none of them has been considered unsatisfactory, while only one was excellent, in the Population attribute. We also see some diversity with regard to the performance of the health units, seeing that they are classified either as reasonable or good.

In the North District, a health unit got reasonable values in four of the five attributes assessed, except in Support Systems, which were considered good at the unit. We can also see that, as a rule, the PHC territories showed a good result in most attributes, such as Population (*n* = 37; 80.4 %), Primary Health Care model (*n* = 43; 93.5 %), Support System (*n* = 45; 97.8 %); however, in relation to the Logistic System (*n* = 32; 76 %) and Governance System, a smaller number of territories showed this classification (*n* = 31; 67.3 %).

**Fig. 3:**
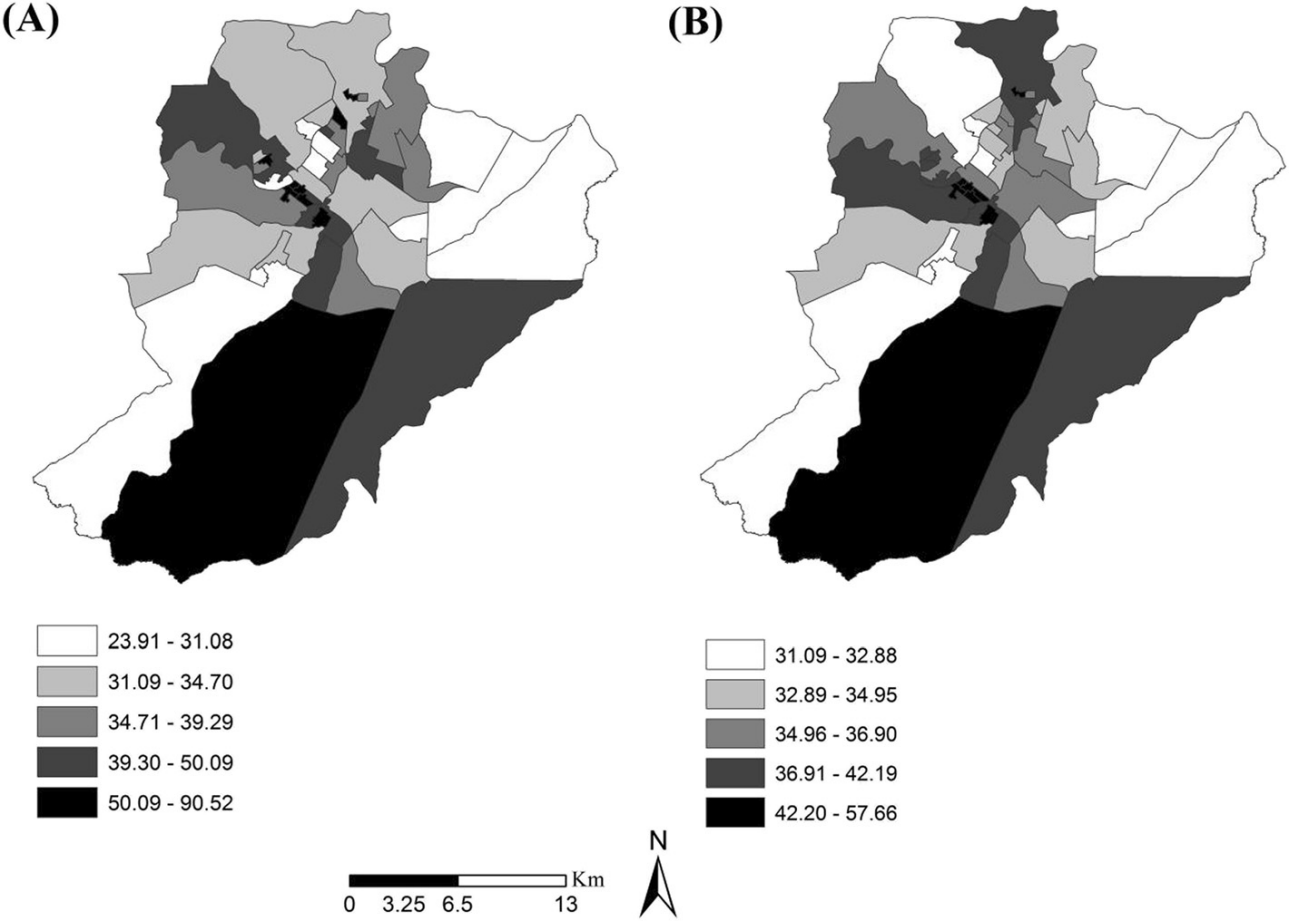
Coropletic map of raw rates (**a**) and Bayesian rates (**b**) for TB avoidable hospitalizations, Ribeirão Preto, São Paulo, Brazil (2006–2012)

Figure 3 shows the maps constructed by considering local raw and Bayesian rates for avoidable hospitalizations, and here we can see a slight mitigation on the second map; however, most of the territories have not shown significant changes when it comes to the annual rates.

The darker areas have shown themselves to be more critical, in relation to the occurrence of hospitalizations and, after the due mitigations, these rates varied between 42.20 to 57.66 cases per 100,000 people, with most of the areas of the East Zone being in this condition. We have also seen some critical areas in other Health Districts, but to a lesser degree.

In a comparative analysis between Figs. 2 and 3b, we see that in the case of the Population attribute, the territories of the PHC units considered as being of regular condition had rates ranging from 34.96 to 36.90 cases per 100,000 inhabitants. The Logistic System attribute had the largest number of areas considered as being of regular condition, and their rates ranged between all the values obtained (32.89 to 36.90).

In relation to the Support System, many of the territorial areas in reasonable condition posted rates ranging from 32.89 to 34.95 cases per 100,000 people. The Governance System attribute also showed a considerable number of areas with reasonable conditions, but the rates here ranged from 32.89 to 57.66 cases per 100,000 inhabitants.

Using the model of multiple linear-regression, we saw no evidence of a statistically significant connection between the Health Care Networks and avoidable hospitalizations for TB (R2 = - 0.003118; *p* = 0.4465).

## Discussion

### Main findings of this study

The purpose of this study was to investigate which territorial areas and which attributes have presented a greater advancement of PHC, in terms of coordination of the HCNs, and also to check if, in critical areas of PHC with regard to coordination, there have been more cases of TB avoidable hospitalizations.

The results show that, as a rule, the PHC has shown good conditions for the coordination of the HCNs, but has shown itself to be weak in relation to the Logistic Systems and Governance Systems attributes.

Regarding the rates of avoidable hospitalizations, in the municipality studied we observed annual rate of 26.2 cases per 100,000 people, a rate which is well above the national mean of 7.2 cases of hospitalizations per 100,000 people, and also above the rate of the State of São Paulo, which came to 4.2 cases per 100.000 inhabitants [31].

In a comparative analysis, comparing results with those from other countries like China, also on the list of the 22 countries that account for 80 % of TB cases in the world, we see that the rates of avoidable hospitalizations are less, as in that country, according to a study, practically 91 % of all TB cases diagnosed had prior hospitalization [32], which has also been made evident in other countries in Asia and Africa [33]. One issue that must be made evident is that many countries have found difficulties to overcome the hospital-centred model, moving to that of Primary Health Care, ordering the Networks [34].

Studies show that when the PHC is of high quality and has the capacity to co-ordinate the Health Care Networks (HCNs) [8]. It also has the capacity to significantly reduce avoidable hospitalizations [35, 36]; however, the study showed no evidence of this relationship.

In addition, we can see important variations in PHC, when it comes to its capacity of co-ordination, which can be justified by the diversity in relation to the model of attention, seeing that some units have made more progress regarding the attributes of coordination, and others less. It is also important to highlight that the process for the establishment of an attention model does not follow linear logic and is, first and foremost, a vector resulting from external forces (for example, quality of human resources) of the health policies inherent to the system itself, such as adjacent social values, community movements, material and non-material resources, and the education of workers and of the community [37]. Therefore, these are forces that partly overlap and result in the performance of the unit, which could explain the differences which have been found in the results of the study.

Another point to be considered is the lack of proportion in the size of the PHC territorial units, with some being very small and some relatively larger, which could also have had an effect on the performance of health units. The larger areas are entirely made up of traditional Basic Health Units (UBS), in which the logic for offer of actions is based on the programmed agenda and spontaneous demand of the users, different from the Family Health Strategies (FHSs), essentially based on the clustering of the clients, which is an important tool when one seeks to establish the coordination of a system [38].

### What is already known about this topic

It must also be mentioned that Brazil has amassed, in its track record, some experiences with the traditional model, and one of the challenges that now looms is that of the conversion of this model to FHS [39, 40]. One must also mention the nationwide effort, through inductive and encouraging policies, to obtain the implementation of FHS as an alternative care model which is more focused on the management of the disease; however, some impasses are still significant, such as the settlement of professionals, the demand for establishment of career, post and salary plans, and also the Law of Tax Responsibility, making the management of municipalities more difficult, in the attainment of this goal [41].

The East District, which includes the more socially affluent regions of the municipality, was the sector which showed the highest number of hospitalizations, and one of the reasons considered to explain this fact was related to the peripheral areas wrapped around the condominiums of the more wealthy segments of the population. Here we stress that, in these areas, there is a low percentage of people who are dependent on the Unified Health System (SUS), and this may somehow interfere in accessibility of health services [42]. In addition, this district has good coordination conditions for most attributes and globality of PHC.

We can also see that the North District has shown some critical areas when it comes to avoidable admittances to hospital, but, in a situation different from that in the East District, its territories have a reasonable condition in relation to the HCNs. This District has the lowest Municipal Human Development Index (MHDI) when compared to the others, and also has a higher demographic density per household, the largest number of subnormal clusters and the highest percentage of people who are exclusive users of the SUS [42]. One explanation of the result found refers to the non-availability of tools within the scope of PHC to cope with social problems that recur in the territory, such as violence, drugs, extreme poverty, educational problems, unhealthy living conditions, and unemployment, among others [43].

Similarly, in Latin America, another challenge that looms is that of overcoming the conception of PCH as a level within a health system, or even a focus on the less affluent segments of the population, with improvised funding and low professional qualification. This context is widely different from that of most European countries, which have implemented PHC as a coordination axis for a system, and universal coverage for all segments of the population, regardless of economic or social status [44, 45]. This means that the main challenge for the 21^st^ Century is the very institution of PHC, in an expanded and wide-scope perception, as recommended by PAHO [46, 47].

It shall also be mentioned that the solution for the crisis of the health systems throughout the world is now betting on a new logic, that of organising them under the control of PHC [7], which PAHO sees as an innovative development in Latin America [46]. In the opinion of Mendes [21], only PHC has the capacity of coordinating the system and catering to 85 % of health needs, with this institution also having the responsibility of regulating users through the Health System and, for this, there is a need for instruments and also for the innovation of the processes [48, 49].

### What this study adds

Several authors [10, 46, 50–52] consider the weight that the chronic conditions have upon global health, and diseases such as TB cannot be countered with efficiency, effectiveness and solution ability through Health Systems that are organized in a fragmented manner, as not only does this produce diseases resulting therefrom, but this is not cost-effective and causes a lot of human suffering.

Based on this context, the study can help towards making a situational diagnosis of PHC and also show relevant aspects for the establishment of a system in HCNs. The municipality did not achieve a classification of excellent in relation to the integration of its system, but may take a step in this direction, provided there is a review of some aspects such as the Logistic System itself, through the implementation of electronic files and computerized systems which allow the PHC to coordinate the users through the health system. It is important to introduce a unique and integrated communication system that allows fluid connection between different points of attention, or even with the introduction of systematic devices for effective communication between the systems that are already in existence.

Another possibility is an investment in the transport system, thereby allowing the user to move between different points of attention. It is therefore seen that the responsibility for health does not rest only with the user, but is shared with PHC, as a party that is co-responsible for the health of the territory, thereby providing all the means and resources so that this user may access the different health services within a HCN, from the perspective of integrality of actions.

The Logistic Systems, in turn, are presented by PAHO [46] as being technological solutions for the transformation of local health systems into HCNs, but these have strong influence of the current health care model, considering that if the work logic in a certain unit is not open to innovations, no matter how many tools are introduced, then it is highly unlikely that they shall have any impact on the supply and the production of their actions.

The study by Onocko-Campos [53] which aimed at appraising the innovative strategies of PHC, brings as new challenges the implementation of new production modes in the health area and also the investment in technologies that allow the coordination and monitoring of the user with chronic illnesses, during the course of time. Nodaria et al. [48] also presents, in their study, the innovation of PHC, bringing interesting findings and results, on introduction of a registration and information system, such as better acceptance, speed of service, and user satisfaction.

The introduction of a Logistic System is part of a rational and honest organization of flows and counterflows of information, products and people at the HCNs, thereby favouring a system which is effective with regard to reference and counterreference throughout the health care points and Support Systems [10, 46, 51, 52, 54–56].

Governance is another issue that has been made evident in the study, with it being of great value to set a mission and also common goals between the different players and managers who are part of the system, so as to establish a cooperative network and also to complement actions, seeking integrality and equity in the supply of health services. Another important investment when the consolidation of a HCN is sought is the reinforcement of the process of regionalization with solidarity, between the Health Districts and even between territorial bases, as the idea of working as a Network makes it somewhat possible to have homogeneity regarding the performance of the units, as these are then developed almost in an independent and cooperative way [10].

In addition, the analysis by attributes of the instrument has allowed the identification of the poorest-performing territorial areas, when it comes to coordination. Albeit in lesser quantity, some areas have had their units joined together under regular conditions in the attributes of Population, PHC and Support Systems; Moreover, in these areas there were distinct situations regarding hospitalizations for TB that were considered avoidable, meaning that it is not possible to establish any kind of relationship between the events.

One possible explanation for the result is due to the centrality of the attention for TB in reference outpatient departments, and also the non-sharing of responsibilities between the reference units and PHC [56–60], which ends up interfering with the results of the study. However, it is important to mention that it is the responsibility of PHC to carry out actions for promotion of health and prevention in the health sector, and within the perspective of HCNs, it would be the responsibility of the PHC to coordinate these patients, this being a point that needs to be reviewed by the management team and in which further studies are necessary.

In relation to the analysis of the cases of TB avoidable hospitalizations, there was the mention of some advantages of application of local empirical Bayesian rates with regard to the raw rates. The Bayesian rates showed lesser variability and a greater adjustment to the real risk of occurrence of the event in each area of the region under study. For those areas where the raw rate would show significant variability, the local empirical Bayesian method has allowed a special effect on the estimates, bringing them closer to a local mean, which generated certain mildness in the thematic map.

Here, it is worth adding that no other study has appraised the HCNs coordinated by the PHC considering the technologies for spatial analysis, possibly with this being a relatively new issue in Brazil. This technology has showed itself to be really interesting in that it maps a social reality of the PHC units and also shows the territorial areas that are most critical. From the standpoint of management, these areas could be prioritized in terms of sanitary investment and planning; within a proposal of establishment of a Network, this study points to areas that are of priority importance for the project.

### Limitation of the study

Due to the ecological approach, there are some limitations inherent to this kind of study, such as ecologic fallacy or bias, which renders it impossible to transport the findings to the individual reality [61].

Despite a probabilistic design of the sample, there are some variables which do indeed interfere with the generalization of the sample for the population of processional people, such as the turnover of professionals, holiday entitlements, sick leave, acceptance of the study, and others. In one health unit, there was a rejection of about 30 % of the team of professionals, especially within the medical category. One strategy that was considered by the authors was that of an articulation with the managers of the services and the coordinators of the Health Districts, which made interaction with the professional staff easier.

To this, we also add, as an additional limitation, the use of secondary data, such as subnotifications and incomplete information which [62], as a result, may not be representative of the real incidence of available hospitalizations within the municipality studied. In addition, it was noticed that, in critical areas of PCH related to the performance of coordination and the occurrence of avoidable hospitalizations, there was no evidence that would confirm this relationship, meaning that there could be other variables to explain these hospitalizations and which interfere with accessibility; elements such as poverty, social exclusion, malnutrition, and other documents determining access [63]. Furthermore, in the study there was the use of one single instrument using closed questions, to assess different models of PHC, which are immersed in singularities and subjectivities of the professional people, and this, to a certain extent, could have brought a certain bias to the study. For any future investigations, it would be an interesting proposition to carry out qualitative studies to identify possible explanations for avoidable hospitalizations for TB, as this could not be explained with a quantitative diagram.

## Conclusion

The study showed the problem of avoidable hospitalizations for TB in an endemic city in Brazil, noting a random distribution of hospitalizations regardless of the quality of the PHV in the co-ordination of Health Care Networks (HCNs).

In terms of the quality of PHC, it was observed that, as a rule, most of the units were within satisfactory standards; however, two of the attributes here appraised were considered as only regular, namely Logistics Systems and the System of Governance. In relation to the Logistics Systems, we were able to observe the lack of an effective system for reference and counter-reference of people and information, throughout the health services offered within the Health Care Networks (HCNs). In relation to the Governance System, the lack of co-operative actions between the different health services and also the lack of definition of a single mission and target, related to the supply of, and actions within, health services.

Through GIS technology, it has been possible to identify the PHC areas that are most deficient with regard to these aspects, thereby contributing with local management in the mapping of critical areas and/or with a more fragile PHC, collaborating towards planning and promotion in the health sector which is better guided, so as to make progress in the improvement of quality and the strengthening of a health service offered by PHC.
